# How Does *PLoS Medicine* Manage Competing Interests?

**DOI:** 10.1371/journal.pmed.0020088

**Published:** 2005-03-29

**Authors:** The PLoS Medicine Editors

## Abstract

PLoS Medicine was launched at a time of unprecedented concern about the influence of hidden competing interests on the medical literature. What is the journal doing to promote transparency, and is our strategy going far enough?

P*LoS Medicine* was launched at a time of unprecedented concern about the influence of hidden competing interests on the medical literature. As a new journal, we had the opportunity to help create “the fully transparent world that is desirable” [1]. What are we doing to promote transparency, and is our strategy going far enough?

We ask all authors and reviewers to declare any competing interests—financial, personal, and professional [2]—and authors' declarations are included with all published articles. We reject articles when we believe that authors' competing interests have compromised their work; for research articles, this means compromised in either the conduct of a study or its interpretation. When we are concerned that reviewers' competing interests may prevent them from giving an unbiased assessment, we find alternative reviewers. And, as recommended by the International Committee of Medical Journal Editors [3], we decline to publish studies when the sponsor controls the decision on publication.

Our main strategy for managing competing interests is disclosure. Financial relationships between industry, researchers, and academic institutions are widespread [4], and disclosing these competing interests is a crucial step in helping to protect the public and the reputation of authors and of *PLoS Medicine*. Disclosure also matters because there is increasing evidence that authors' competing interests have a strong influence on their conclusions. For example, review articles looking at the scientific evidence on the health effects of passive smoking have reached different conclusions; a study of these review articles found that the only factor associated with the conclusion that passive smoking was harmless was whether an author was affiliated with the tobacco industry [5]. A study of 159 randomized clinical trials found a significant association between authors' financial competing interests and their favorable conclusions about an experimental intervention [6].

We cannot rely entirely on peer review to detect bias in the conclusions of an article, because although peer review may help to uncover some types of bias, such as bias in the study design, it cannot detect other types, such as bias in the study's conduct. Because a competing interests statement accompanies every article published in *PLoS Medicine*, readers can take these interests into account when they assess a paper themselves. The reality is that readers are wary of competing interests. In one study, readers were randomly sent the same paper with or without

Financial relationships between industry, researchers, and academic institutions are widespread.

the authors having disclosed a financial competing interest [7]. Readers scored the paper with the competing interest significantly lower on all five measures: interest, importance, relevance, validity, and believability of the study.

Our policy of asking authors to disclose their competing interests is not, of course, foolproof. The Center for Science in the Public Interest recently examined 163 articles in four scientific journals and identified at least 13 articles for which authors did not disclose relevant conflicts of interest that should have been disclosed according to the journals' policies [8]. Bero and colleagues compared the statement of competing interests of the authors of a 2003 *BMJ* paper on the health effects of secondhand smoke with internal tobacco industry documents describing financial ties between the industry and the authors [9]. Although the authors met the *BMJ*'s requirements for financial disclosure, the disclosure did not provide readers with a full picture of the industry's long-standing involvement with the authors.

Should we as journal editors be investigating authors' ties for ourselves? We do not have the resources to do so, and agree with Bero and colleagues that “an elaborate policing operation is not feasible or necessarily desirable” [9]. But in this particular case, say the authors, a quick search of the tobacco documents that are freely available online (e.g., at http://www.legacy.library.ucsf.edu or http://www.bat.library.ucsf.edu) would have been revealing. Maybe we are heading towards an era when such searches become more common—perhaps initially by randomly selecting papers for investigation.

At last year's Council of Science Editors retreat on competing interests, editors came up with a list of questions to think about when formulating a journal's competing interests policy (Table S1). Some are straightforward. Should editors declare their own competing interests? We think so (and have declared ours at http://medicine.plosjournals.org/perlserv/?request=get-static&name=editors_interests). Others are more complex. When editors discover that a published author failed to declare a significant competing interest, what should they do? Should they impose sanctions on the author? Should they publish a correction or even retract the paper?

To help us answer such questions, and to advise us on individual cases for which we are concerned about competing interests or broader ethical questions, we have appointed an external advisory group. The group (Table S2) has expertise in clinical medicine, medical editing, research, health policy, law, and bioethics, and includes a lay member. In addition, an internal committee at PLoS meets monthly to consider competing interests across the organization. We are taking this issue seriously, because we recognize that journals are seen as the gatekeepers of published research. We welcome your feedback on how we are doing at protecting the probity of our content.

## Supporting Information

Table S1 Competing Interests Policies: Questions for Editors to Consider (33 KB DOC).

Table S2 Members of the *PLoS Medicine* Advisory Group on Competing Interests and Publication Ethics (28 KB DOC).
