# Supplementary material for: How Does PLoS Medicine Manage Competing Interests?
Source: PLoS Med. 2005 Mar 29;2(3):e88. doi: 10.1371/journal.pmed.0020088 (PMC1069676; doi:10.1371/journal.pmed.0020088)
Supplement: Table S1 — (33 KB DOC). [file pmed.0020088.st001.doc]

**Table S1.** Competing Interests Policies: Questions for Editors to Consider

Editors attending the Council of Science Retreat on *Conflicts of Interest and Scientific Publication*, October 29–31 2004, compiled a list of questions for journals to consider in formulating a competing interests policy.

Authors’ Competing Interests

▪ Does your journal have a competing interests policy?

▪ When was it developed?

▪ Who developed it? The publisher? The society?

▪ When did you last review it?

▪ Does the policy include a definition of a competing interest?

▪ Does the policy address financial interests only, or does it include other types of competing interests (such as personal/professional relationships, non-paid consultancies, ideology etc.)?

▪ Are examples of competing interests provided?

▪ Does the policy define what relevant/important interests means?

▪ For financial interests, do you give a dollar limit or other types of limit (e.g. duration of time of a consultancy)?

▪ What happens when authors declare competing interests: who are these disclosed to? Are they disclosed to news media (e.g. in press releases)? How do you manage competing interests (e.g. do you write to the dean of the school asking for confirmation)?

▪ Should the lead author answer competing interests questions for all authors?

▪ If authors have no competing interests to declare, do you state this?

▪ Is the policy voluntary, encouraged, or required?

▪ If required, at what stage do you ask about it (manuscript submission, revision, or acceptance)?

▪ For non-compliance (i.e. when an author has failed to disclose an important competing interest), what do you do? Do you prohibit the author from publishing again in your journal? Do you report the problem to the author’s dean? Do you publish a correction? Do you retract the paper? Do you “name and shame” the author in the journal? If your journal has a policy on publishing a correction or naming and shaming, do you state this in your policy?

▪ How is the policy communicated to authors? Do you put the policy in your instructions to authors? If so, what does it include? Is there a disclosure form? If there is, how detailed is it? Are you asking about dollar ranges? Are you writing editorials about your policy? Do you discuss competing interests in your decision letters to authors?

▪ Does your policy apply to all articles or only certain types?

▪ Will disclosure have any bearing on the chances of acceptance?

▪ Should there be different thresholds of disclosure for different types of article (e.g. editorial vs. review article vs. research vs. poem)?

▪ Funding sources: do you have a policy for authors on declaring funding sources? Does it include non-monetary support (e.g. in-kind donations)? Does the journal have a policy that authors should describe the role of the funding source in the work to be published (e.g. “the funder supplied materials but did not own the data”)?

Access to Data

▪ Does the journal have a policy on access to data?

▪ Does it address data sharing and depositories? Trial registries?

▪ Does it address who has access to the data reported?

▪ Is it voluntary or required?

▪ Is it communicated to authors?

Competing Interests of Reviewers, Editors, Editorial Boards, Boards of Directors, Journal Publishers/Owners

▪ Do you have policies on competing interests for all of these groups?

▪ Have you made it clear to reviewers that they shouldn’t use the data for their own personal financial gain?
