# Supplementary material for: How Does PLoS Medicine Manage Competing Interests?
Source: PLoS Med. 2005 Mar 29;2(3):e88. doi: 10.1371/journal.pmed.0020088 (PMC1069676; doi:10.1371/journal.pmed.0020088)
Supplement: Table S2 — (28 KB DOC). [file pmed.0020088.st002.doc]

**Table S2.** Members of the *PLoS Medicine* Advisory Group on Competing Interests and Publication Ethics

▪ **Lisa Bero** (Chair of the Advisory Group), Professor, [Department of Clinical Pharmacy](http://www.ucsf.edu/clpharm/index.htm), School of Pharmacy and Institute for Health Policy Studies, School of Medicine, [University of California San Francisco](http://www.ucsf.edu/), United States. http://itsa.ucsf.edu/~tobacco/lisa.htm

▪ **Carl Elliot**, Associate Professor, Center for Bioethics; Department of Pediatrics, University of Minnesota Medical School; Department of Philosophy, University of Minnesota, United States. <http://www.bioethics.umn.edu/faculty/elliott_c.shtml>

▪ **Sheldon Krimsky**, Professor of Urban & Environmental Policy & Planning, Tufts University, and Adjunct Professor in the Department of Family Medicine and Community Health, Tufts Medical School, Medford, MA, United States. http://www.tufts.edu/~skrimsky/

▪ **Michelle Mello**, Assistant Professor of Health Policy and Law, Department of Health Policy and Management, Boston, MA, United States. Harvard University. http://www.hsph.harvard.edu/faculty/MichelleMello.html

▪ **Walter Newman**, (Lay Member of the Advisory Group), Lay Member of the University of California Conflict of Interest Advisory Commitee

▪ **Jerome Singh**, Head of the Bioethics and Health Law Programme at the Centre for the AIDS Programme of Research in South Africa (CAPRISA), Nelson R. Mandela School of Medicine, Durban, South Africa. http://www.caprisa.org/People/cv/jerome.html

**▪ Michael Wilkes**, Professor of Medicine and Vice Dean of Medical Education, University of California Davis, United States
